# Bee (*Apis mellifera* L. 1758) wax restores adipogenesis and lipid accumulation of 3T3‐L1 cells in cancer‐associated cachexia condition

**DOI:** 10.1002/fsn3.4153

**Published:** 2024-04-17

**Authors:** Hyun‐Jun Jang, Hyun‐Yong Kim, Ji Hyo Lyu, Subramanian Muthamil, Ung Cheol Shin, Hyo Seon Kim, Jieun Jeong, Suwhan Chang, Yun Kyung Lee, Jun Hong Park

**Affiliations:** ^1^ Herbal Medicine Resources Research Center Korea Institute of Oriental Medicine Naju Korea; ^2^ Research Group of Personalized Diet Korea Food Research Institute Wanju‐gun Korea; ^3^ Laboratory of Integrative Oncolomics, Department of Biomedical Science, College of Medicine University of Ulsan Seoul Korea; ^4^ Korean Convergence Medicine Major University of Science & Technology (UST) Daejeon Korea

**Keywords:** adipocytes, adipogenesis, bee wax, cancer‐associated cachexia, lipolysis, oxygen consumption

## Abstract

Cachexia is associated with various diseases, such as heart disease, infectious disease, and cancer. In particular, cancer‐associated cachexia (CAC) accounts for more than 20% of mortality in cancer patients worldwide. Adipose tissue in CAC is characterized by adipocyte atrophy, mainly due to excessively increased lipolysis and impairment of adipogenesis. CAC is well known for the loss of skeletal muscle mass and/or fat mass. CAC induces severe metabolic alterations, including protein, lipid, and carbohydrate metabolism. The objectives of this study were to evaluate the effects of bee wax (*Apis mellifera* L. 1758) (BW) extract on adipogenesis, lipolysis, and mitochondrial oxygen consumption through white adipocytes, 3T3‐L1. To achieve this study, cancer‐associated cachexia condition was established by incubation of 3T3‐L1 with colon cancer cell line CT26 cultured media. BW extract recovered the reduced adipogenesis under cachectic conditions in CT26 media. Treatment of BW showed increasing lipid accumulation as well as adipogenic gene expression and its target gene during adipogenesis. The administration of BW to adipocytes could decrease lipolysis. Also, BW could significantly downregulated the mitochondrial fatty acid oxidation‐related genes, oxygen consumption rate, and extracellular acidification rate. Our results suggest that BW could improve metabolic disorders such as CAC through the activation of adipogenesis and inhibition of lipolysis in adipocytes, although we need further validation in vivo CAC model to check the effects of BW extract. Therefore, BW extract supplements could be useful as an alternative medicine to reverse energy imbalances.

## Graphical abstract

Bee wax extract has a protective effect against cachexia‐associated adipogenesis inhibition. Bee wax extract enhances adipocyte differentiation and lipid accumulation during adipogenesis. Bee wax extract downregulates lipolysis in mature adipocytes and inhibits mitochondrial fatty acid oxidation and glycolysis in mature adipocytes.

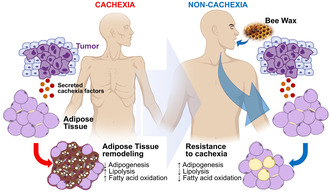

## 1 INTRODUCTION

Cachexia refers to a systemic wasting condition that causes muscle debilitating, accompanied by unintentional weight loss. Cachexia is considered as a late consequence of diseases such as heart disease, infectious disease, and cancer. In the case of cancer, cachexia occurs in over 70% of cancer patients and accounts for more than 20% of mortality in cancer patients worldwide (especially 30–50% of mortality in gastrointestinal cancer patients) (Palesty & Dudrick, 2003). Cancer‐associated cachexia (CAC) is known for body weight loss (10% or more within 6 months), especially adipose tissue and skeletal muscle weight with chronic inflammatory conditions (Lee et al., 2022). Particularly, adipose tissue atrophy is affected by various metabolic complications. Although CAC is a well‐studied multi‐organ crosstalk, mechanisms of adipose tissue loss have recently been explored, and many concepts remain to be determined (Sun et al., 2020). Generally, adipose tissue in CAC is characterized by adipocyte atrophy, mainly due to high levels of lipolysis and modification of the extracellular matrix, resulting in impairment of adipogenesis, fibrosis, dysfunction of energy metabolism, and chronic inflammation (Sun et al., 2020; Yang & Mottillo, 2020). Consequently, remodeled adipose tissue is able to secrete free fatty acids and adipokines in addition to inflammatory cytokines. Adipose tissue consists of heterogeneous cell types such as adipocyte precursor cells, including stromal populations, mature adipocytes, immune cells, vascular cells, lymph nodes, blood vessels, and nerve cells. Loss of adipose tissue in CAC has been explained by increased lipolysis due to lipid mobilization and impaired adipogenesis in adipose tissue (Daas et al., 2018).

In general, adipocytes are mainly involved in the storage of excess fatty acids (FAs) in lipid droplets (LDs) in the form of triacylglycerols (TAGs). During energy‐demanding status, stored TAGs are breaking into FAs and glycerol. Adipogenesis is the process of the formation of mature adipocytes from undifferentiated preadipocytes (Jung et al., 2023). Also, adipogenesis is a well‐known multi‐step cascade requiring specific transcriptional factors, including peroxisome proliferator‐activated receptor γ (PPARγ) and CCAAT/enhancer‐binding proteins (C/EBPs) such as C/EBPα, C/EBPβ, and C/EBPδ. Upon induction of adipogenesis, C/EBPβ and C/EBPδ levels are increased and subsequently induce the expression of PPARγ and C/EBPα (Lee, Cho, et al., 2020; Tang et al., 2004). C/EBPα and PPARγ are crucial for terminal differentiation into adipocytes through the induction of adipocyte functional molecules, such as adipocyte protein 2 (aP2), lipoprotein lipase (LPL), perilipin, and fatty acid synthase (FAS). Moreover, lipolysis breaks down TAGs through lipase into FAs and glycerol. The major lipases are hormone‐sensitive lipase (HSL), patatin‐like phospholipase domain containing‐2 (PNPLA2)/adipocyte triglyceride lipase (ATGL), and monoacylglycerol lipase (MGL).

In the aspect of adipogenesis under cachexia, several studies have reported that cachexia has been shown to suppress adipogenesis in in vitro co‐culture and in vivo models (Batista Jr. et al., 2012; Bing et al., 2006; Lopes et al., 2018). To discover natural products reversing the cachectic effect on adipogenesis, we screened natural products and found that bee (*Apis mellifera* L. 1758) wax (BW) restores adipogenesis in cancer‐associated cachexia condition. Treatment of BW on adipocytes was limitedly studied compared to bee venom. However, bee venom has been reported to inhibit adipogenesis in 3T3‐L1 adipocytes and to reduce adipogenesis in high‐fat‐dieted mice (Cheon et al., 2017; Kim et al., 2020). However, the effect of BW on adipogenesis and lipolysis activity under cancer cachectic conditions has not yet been investigated. With this background, the objective of this study was to investigate the effects of BW on adipogenesis under cachectic conditions, lipolysis, and mitochondrial oxygen consumption, using white adipocytes 3T3‐L1 cells.

## 2 MATERIALS AND METHODS

### 2.1 Bee wax extraction

Beehive wax was collected in Yeongwol, Ganwon‐do, South Korea. The collected bee wax was dried and ground to powder. A total of 1 kg of ground bee hive wax powder was extracted by reflux of distilled water at 100°C for 3 h, filtered (53 μm sieve, Sigma‐Aldrich, USA), and lyophilized to get the BW extract powder.

### 2.2 CT26 cachectic‐conditioned media (CCM)

To prepare cachectic‐conditioned media, the previous cachexia induction method was modified and adopted (Chung et al., 2011). Briefly, CT26 cells were seeded in a T175 flask (1 × 10^5^ cells/flask) and cultured in RPMI media (Gibco, Grand Island, NY, USA) consisting of 1% penicillin–streptomycin (P/S) (Gibco, Grand Island, NY, USA) and 10% fetal bovine serum (FBS) (Gibco, Grand Island, NY, USA). After 12 h, RPMI medium was replaced with 30 mL of DMEM (with high glucose, GlutaMAX™ Supplement, pyruvate, Gibco, Grand Island, NY, USA) containing 10% FBS and 1% P/S and incubated for 4 days. After incubation, supernatants were collected, 50 mL of fresh DMEM were added. After another 3 days, supernatants were collected by centrifugation (2000 rpm for 10 min at 4°C) and filtered by a 0.22 μm cellulose acetate filter (Corning, NY, USA).

### 2.3 3T3‐L1 cell culture and adipocyte differentiation

**FIGURE 1:**
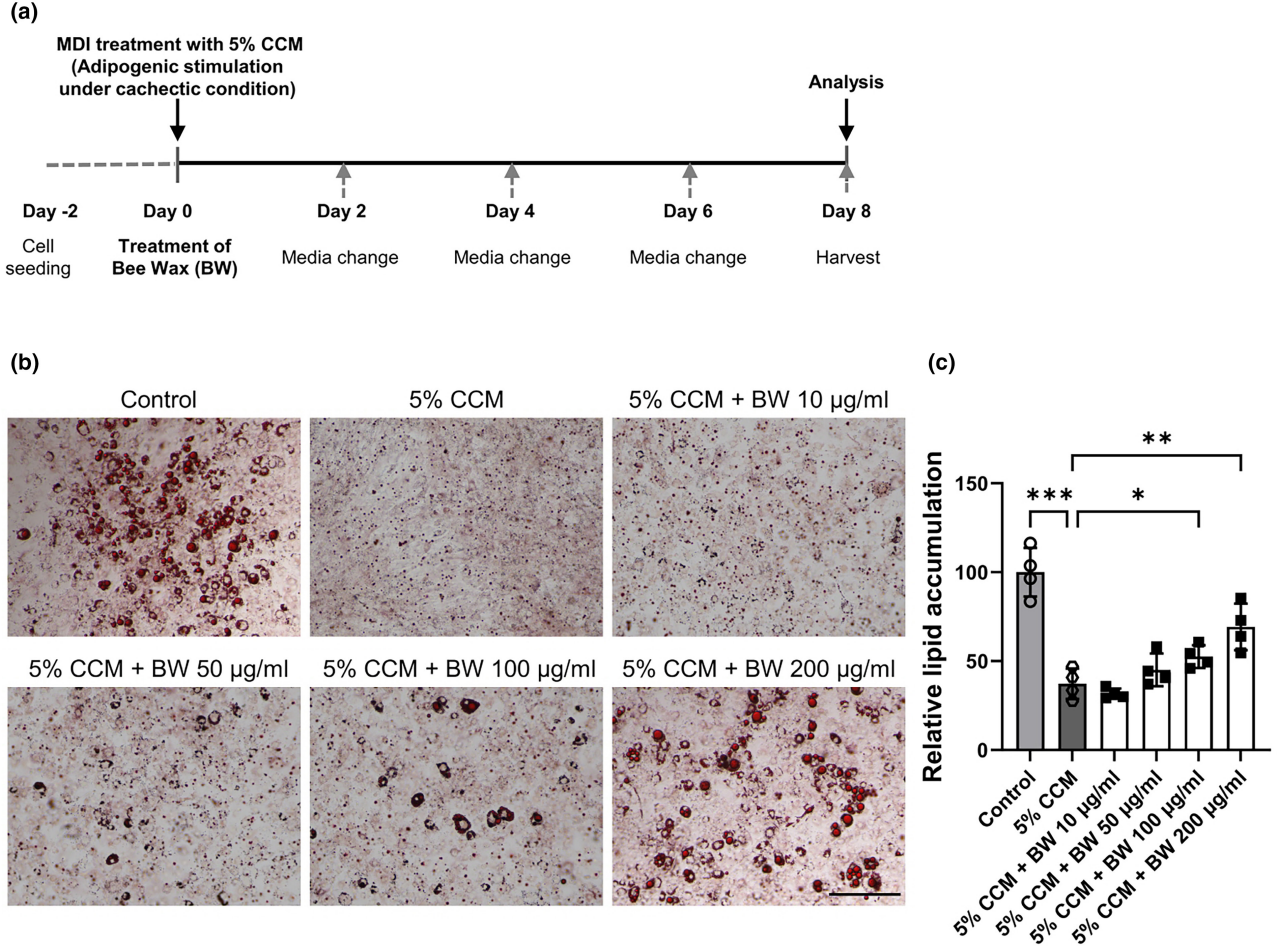
Effects of bee wax (BW) extract on adipogenesis under cachectic condition. (a) Schematic diagram of treatment of BW extraction during adipocytes differentiation of 3T3‐L1 cells under cachectic condition by 5% CT26 conditioned media (CCM). (b, c) Effects of BW extraction on adipogenesis and lipid accumulation under cachectic condition were assessed using Oil Red O staining. Black scale bar, 300 μm. Data represent the mean ± SD. *p < .05, ** *p* < .01, *** *p* < .001 indicate significant differences between groups. The significance was assessed by either the one‐way ANOVA, followed by post‐hoc analysis versus control.

In this study, mouse embryo 3T3‐L1 preadipocytes (ATCC, passage number under 10) were used. DMEM supplemented with 1% P/S and 10% bovine calf serum (Gibco, Grand Island, NY, USA) was used to maintain the 3T3‐L1 cells to reach maximum confluency. To induce adipocyte differentiation, the cells were cultured in DMEM containing 10% FBS, 0.5 μg/mL insulin (Sigma‐Aldrich, USA), 5 μM dexamethasone (Sigma‐Aldrich, USA), and 0.5 mM isobutylmethylxanthine (Sigma‐Aldrich, USA) for 2 days (Lee, Chae, et al., 2023). Then the cells are maintained in differentiation media (DMEM containing 10% FBS, 1% P/S, and 0.5 μg/mL insulin) for 6 days, but the media are changed every 2 days. After the differentiation of 3T3‐L1 cells, vehicle (DMSO) (Sigma‐Aldrich, USA) or BW extract were treated at varying concentrations. To create a cachectic condition, the previous cachexia induction method was modified and adopted (Chung et al., 2011). Cachexia‐associated adipogenesis inhibition was induced with 5% CCM and assessed by measuring lipid accumulation (Figure 1a).

### 2.4 Total RNA extraction and quantitative real‐time PCR

From the differentiated 3T3‐L1 adipocytes, total RNA was extracted using the TRIzol method (Ambion, Carlsbad, CA, USA), and quantitative real‐time PCR (qRT‐PCR) was performed (Lee, Kim, et al., 2020). RNA (1 μg) was converted into cDNA (Thermo Fisher Scientific, Cleveland, OH, USA), and SYBR Green was used to quantify the gene expression (SYBR Green PCR Master mix, Enzynomics, Korea). qRT‐PCR was performed on the QuantStudio 7 Flex system (Thermo Fisher Scientific, Cleveland, OH, USA). The relative gene expression was determined using the comparative CT method (Kim, Kim, et al., 2023), and the mRNA levels were normalized to the housekeeping gene, cyclophilin. The details of the primer sequences are given in Table S1.

### 2.5 Oil Red O staining

Mature adipocytes were fixed with 4% formaldehyde for 15 min at room temperature (RT), and the cells were rinsed with phosphate‐buffered saline (PBS). Then, the cells were stained with 0.3% Oil Red O solution (Sigma‐Aldrich, St Louis, MO, USA) at RT for 1 h to assess lipid accumulation (Nakano et al., 2023). Finally, the cells were washed with distilled water and observed under the light microscope for lipid droplet formation (Evos 5000, Invitrogen, USA). Oil Red O staining was quantified at 520 nm after elution with 100% isopropanol (Lee, Jang, et al., 2021).

### 2.6 Cell viability analysis

To check the effect of BW extract on cell proliferation, a cell counting kit (CCK‐8 kit; Sigma‐Aldrich, St Louis, MO, USA) was used (Yan et al., 2023). 3T3‐L1 cells were sown (3 × 10^3^ cells/well) in 96‐well plates and treated with various concentrations of BW extract (50, 100, 200, and 400 μg/mL), incubated at 37°C for 48 h. Then, cells were incubated with the added CCK‐8 reagent for another 2 h. The cell viability was assessed by the absorbance at 450 nm wavelength using SpectraMax iD3 (Molecular Devices, San Jose, CA, USA).

### 2.7 Lipolysis assay

The effect of BW extract on lipolysis was quantified using a free glycerol reagent (Sigma, USA), as measured by the rate of glycerol release based on the instructions (Lee et al., 2018). Briefly, cells were incubated in DMEM containing 2% FA‐free BSA (Sigma‐Aldrich, USA) with/without 1 μM isoproterenol (Sigma‐Aldrich, USA), a non‐specific β‐adrenergic receptor (β‐AR) agonist. The released glycerol level in supernatants was determined spectrophotometrically by measuring the absorbance at 540 nm with SpectraMax iD3 microplate reader. Pierce BCA protein assay reagent (Thermo Fisher Scientific, Cleveland, OH, USA) was used to normalize the glycerol amount to the total protein levels.

### 2.8 Cellular oxygen consumption rate (OCR)

To check the OCR in mature 3T3‐L1 adipocytes, 3200 cells were sown in XF^*e*^96 microplates (Seahorse Bioscience, Agilent Technologies, Santa Clara, CA, USA). Cells were differentiated into mature adipocytes, as per the differentiation protocols described previously (Lee, Park, et al., 2023). The OCR of differentiated adipocytes was analyzed using a Seahorse XF^*e*^96 extracellular flux analyzer (Seahorse Bioscience, North Billerica, MA, USA). After three times of rinsing with PBS, mature 3T3‐L1 adipocytes were incubated in DMEM with 4 mM glutamine (Gibco, Grand Island, NY, USA), 1 mM sodium pyruvate (Gibco, Grand Island, NY, USA), and 25 mM glucose (Sigma‐Aldrich, USA) for 1 h. Carbonyl cyanide‐p‐trifluoromethoxyphenylhydrazone (Sigma‐Aldrich, USA) (2 μM), oligomycin (Sigma‐Aldrich, USA) (2.5 μM), antimycin A (Sigma‐Aldrich, USA) (0.5 μM), and rotenone (Sigma‐Aldrich, USA) (0.5 μM) were used to determine OCR. The values were normalized to the total protein concentration of mature adipocytes.

### 2.9 Statistical analysis

Data are represented as the mean ± SD. Statistical analysis was performed using GraphPad Prism software version 10.0.0 (GraphPad Software, San Diego, CA, USA). More than two group comparisons were conducted using two‐tailed unpaired Student *t*‐tests, the one‐way ANOVA or the two‐way ANOVA, followed by post‐hoc analysis (Lee, Kim, et al., 2021). Statistical significance was defined as *p < .05, **p < .01, and ***p < .001.

## 3 RESULTS

### 3.1 BW extract has a protective effect against cachexia‐associated adipogenesis inhibition

To investigate the effect of BW extract on adipogenesis under cachexia, we made a cachexia mimic condition with 5% CCM (Chen et al., 2020; Han et al., 2022). Although 5% CCM completely blocked the adipogenesis induced by adipogenic stimulation, BW extract significantly restored the adipocyte differentiation suppressed by CCM (Figure 1b). 10 μg/mL BW extract showed marginal effects, but 50–200 μg/mL BW extract increased adipocyte differentiation in a dose‐dependent manner, which was assessed using Oil Red O staining. Adipogenesis inhibited by 5% CCM was recovered by 12.6%, 24.5%, and 51% at concentrations of 50, 100, and 200 μg/mL, respectively (Figure 1c). This result implies that BW extract has a protective effect against cachexia‐associated adipogenesis inhibition. Based on these protective effects and cytotoxicity data (Figure S1), 50 and 100 μg/mL concentrations of BW extract were chosen for the following mechanistic experiments.

### 3.2 BW extract enhances adipocyte differentiation and lipid accumulation during the adipogenesis of 3T3‐L1 cells

**FIGURE 2:**
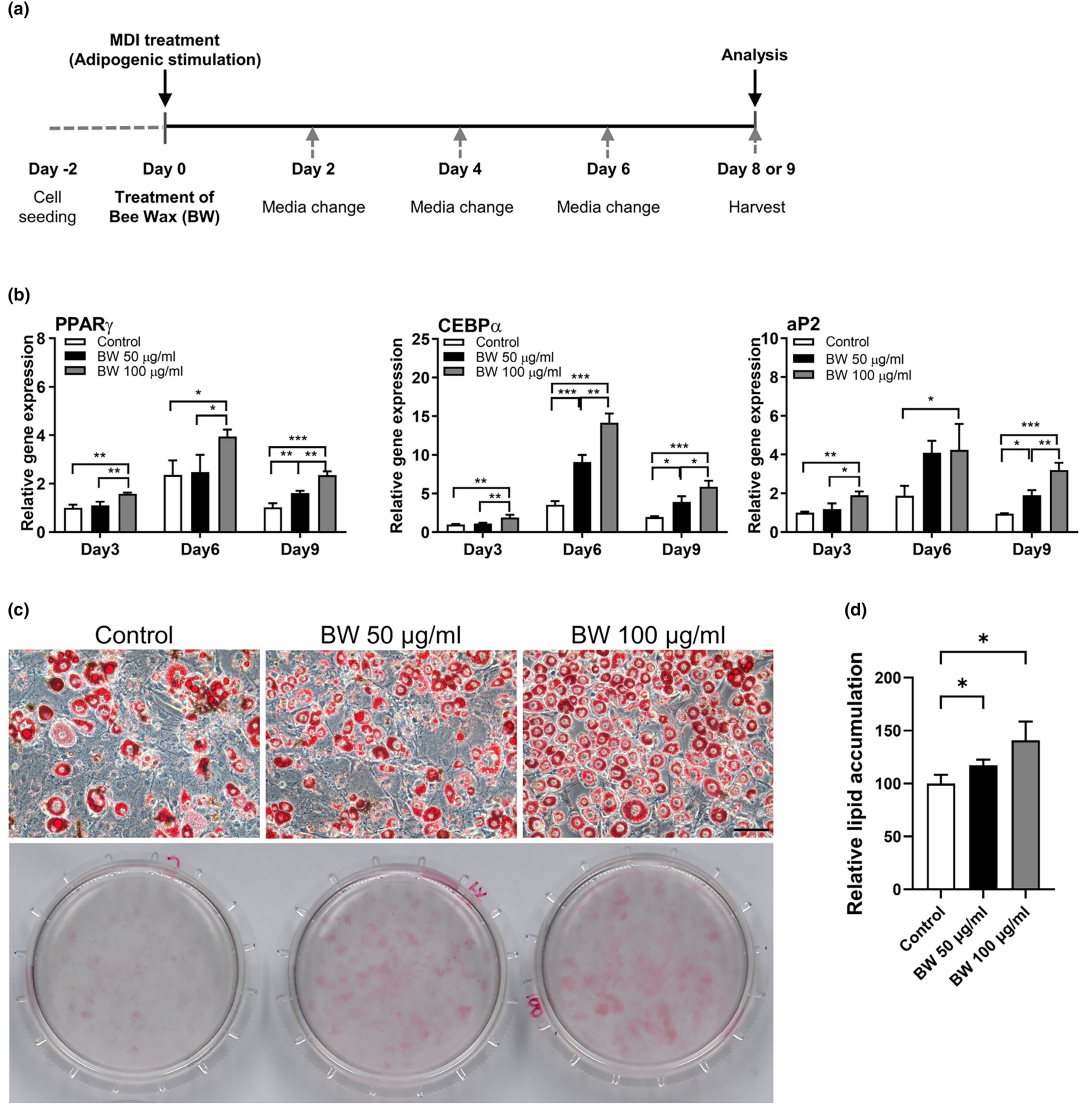
Effects of bee wax (BW) extract on adipocyte differentiation. (a) Schematic diagram of treatment of BW extract during adipocytes differentiation of 3T3‐L1 cells. (b) Effects of BW extract on the expression of adipogenic genes. (c, d) Effects of BW extract on lipid accumulation assessed by Oil Red O staining. Black scale bar, 100 μm. Data represent the mean ± SD. *p < .05, **p < .01, ***p < .001 indicate significant differences between groups. The significance was assessed by either the two‐way ANOVA (b) or one‐way ANOVA (d), followed by post‐hoc analysis.

To explain the protective effect of BW extract against cachexia, we first tested the effect of BW extract on adipocyte differentiation under normal adipogenesis‐inducing conditions. Adipogenesis is regulated by specific transcriptional factors, PPARγ and C/EBPs, which are key regulators of adipocyte differentiation. C/EBPα and PPARγ are well known for acting as mediators for the initiation of adipocyte differentiation through increasing expression of their target genes, such as FABP4 (aP2) and adiponectin (Farmer, 2006; Rosen et al., 2000; Wang et al., 2013). BW extract significantly increased the expression of adipogenic genes from the early stages of adipocyte differentiation to the late stages of adipogenic development (Figure 2b). BW extract considerably increased the expression of C/EBPα, PPARγ, and its target gene aP2, which is a marker of mature adipocyte differentiation (Figure 2b). Additionally, adipocyte differentiation is accompanied by the activation of lipogenesis‐related genes and results in lipid droplet formation (Pantoja et al., 2008). Therefore, the effect of BW extract on lipid droplet formation was tested in differentiated 3T3‐L1 cells. The representative images showed that BW extract dramatically increased lipid accumulation in a dose‐dependent manner (Figure 2c). Adipogenesis was increased by 17.3% and 40.9% at concentrations of 50 and 100 μg/mL, respectively (Figure 2d). These results suggest that BW extract could upregulate lipid accumulation in 3T3‐L1 cells during adipogenesis.

### 3.3 BW extract downregulates the lipolysis in mature 3T3‐L1 adipocytes

**FIGURE 3:**
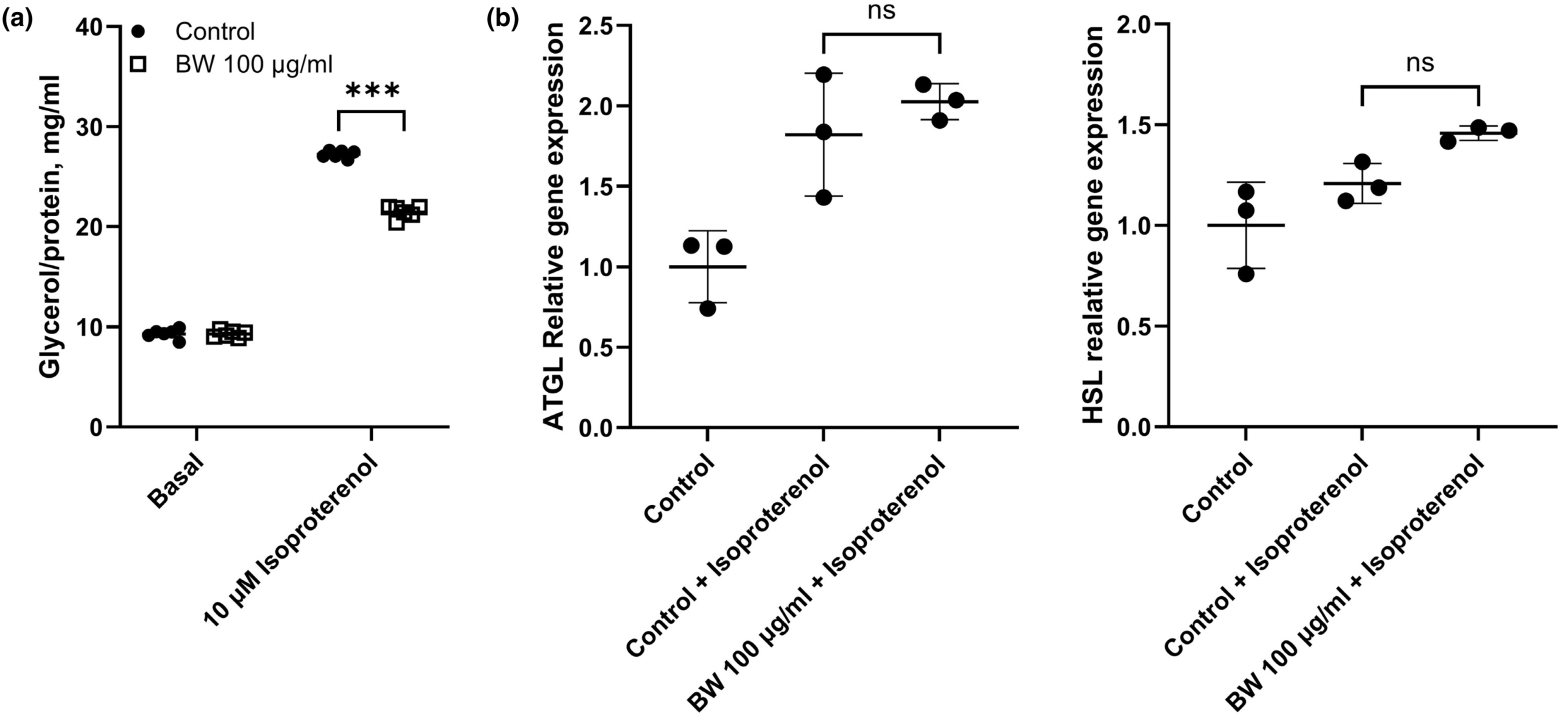
Effects of bee wax (BW) extract on lipolysis and lipolysis‐associated gene expression of 3T3‐L1 cells. (a) Effect of BW extract on lipolysis after differentiation of 3T3‐L1 cells. (b) Effect of BW extract on lipolysis‐related gene expression in mature 3T3‐L1 adipocyte cells. The data represent the mean ± SD. ***p < .01 indicates significant differences between groups and ns indicates not significant. The significance was assessed by either the two‐way ANOVA (a) or the one‐way ANOVA (b), followed by post‐hoc analysis.

The main function of adipocytes is to store excess FAs as TAGs in the cytoplasmic organelles called lipid droplets. When energy demand is increased, stored TAGs are broken down into FAs and glycerol, a process known as lipolysis. The canonical pathway of lipolysis in adipocytes is involved in three major lipases, such as ATGL, HSL, and MGL (Yang & Mottillo, 2020). Most studies have reported dysregulation of lipolysis in adipocytes in metabolic disorders such as obesity, diabetes, liver disease, and cancer. Especially, regulation of lipolysis in adipocytes through oriental medicine could be applied to the treatment of CAC in adipose tissues. Therefore, the effects of BW extract on lipolysis and its related genes in differentiated 3T3‐L1 adipocytes were tested in the present study. Lipolysis of completely differentiated 3T3‐L1 adipocytes was facilitated by isoproterenol as a non‐specific β‐AR agonist. During adipogenesis, no significant changes were observed in basal lipolysis level with BW extract treatment compared to control. Whereas, BW extract significantly reduced isoproterenol‐induced lipolysis by 32.1% (Figure 3a). These results were further confirmed by qRT‐PCR; treatment of BW extract with isoproterenol did not show any significant inhibition in the expression of lipolysis‐associated genes ATGL and HSL (Figure 3b). Taken together, the results imply that BW extract could inhibit lipolysis in mature 3T3‐L1 adipocytes without making any changes in lipolysis gene expression.

### 3.4 BW extract inhibits mitochondrial fatty acid oxidation and glycolysis in mature 3T3‐L1 adipocytes

**FIGURE 4:**
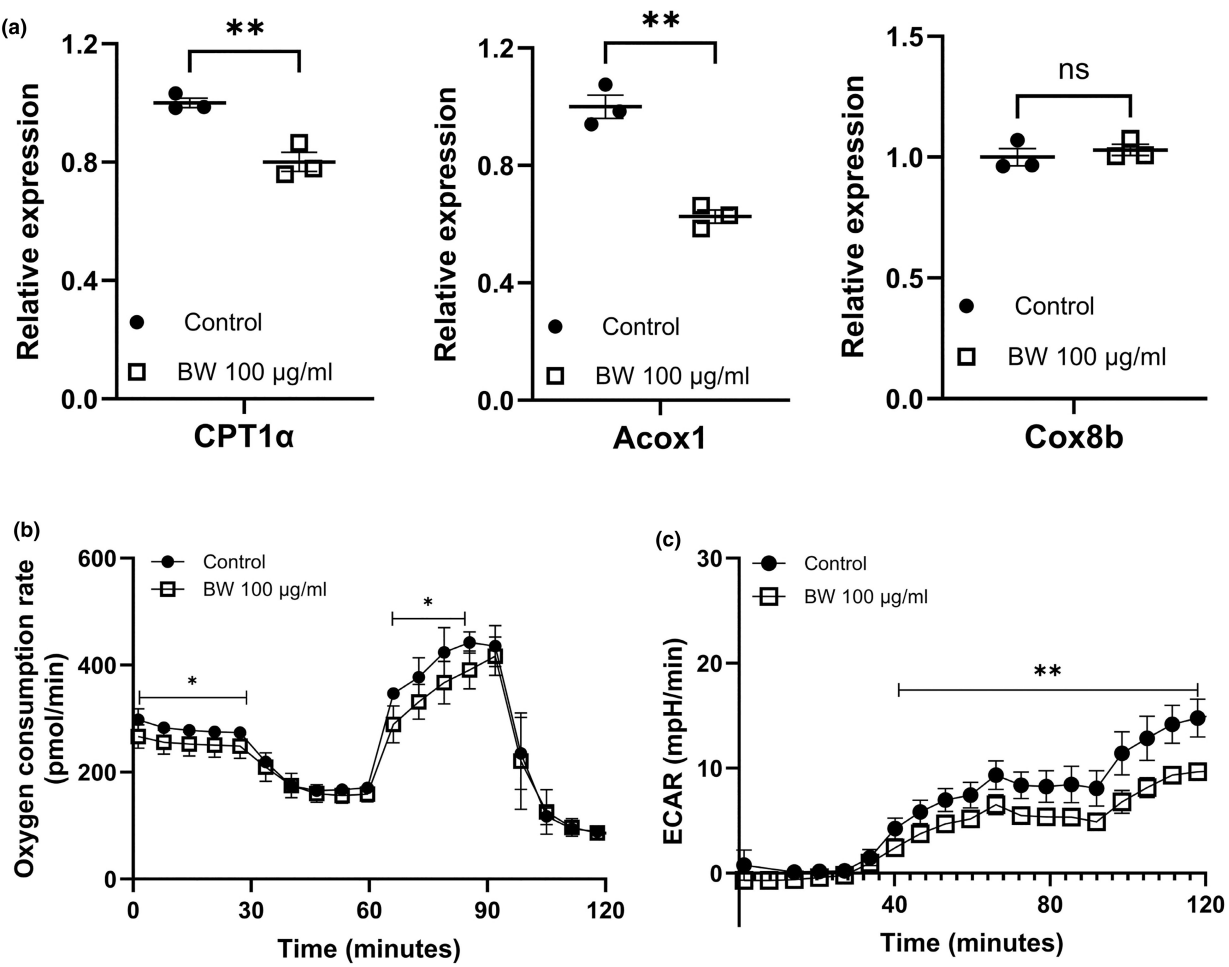
Effects of BW extract on mitochondrial OCR and ECAR in 3T3‐L1 cells. (a) Effects of BW extract on mitochondrial fatty acid oxidation‐related gene expression in fully differentiated 3T3‐L1 cells. (b) Oxygen consumption rate (OCR) of mature 3T3‐L1 adipocytes with the treatment of BW extract. (c) Extracellular acidification rate (ECAR) of 3T3‐L1 cells with the treatment of BW extract. The data represent the mean ± SD. *p < .05 and **p < .01 indicate significant differences between groups, and ns indicates not significant. The significance was assessed by the two‐tailed unpaired Student *t*‐tests.

Upon upregulation of lipolysis, metabolites such as FAs and glucose could be provided as substrates for mitochondrial activities. So, the effect of BW extract on mitochondrial oxidation‐related gene expression in fully differentiated 3T3‐L1 adipocytes was examined. The expression of mitochondrial fatty acid oxidation‐related genes CPT1α and Acox1 was significantly downregulated by 19.9% and 31.4%, respectively, by 100 μg/mL BW extract (Figure 4a). Then, the efficacy of BW extract on mitochondrial OCR in mature adipocytes was evaluated. BW extract at 100 μg/mL concentration significantly reduced mitochondrial OCR by 9.54% and 11.39% in basal and maximal respiration, respectively (Figure 4b). In addition to mitochondrial fatty acid oxidation, the extracellular acidification rate (ECAR) is a measure of lactic acid levels and indicates the conversion of glucose to lactate during glycolysis. The ECAR level also decreased by 35.4% upon BW extract treatment (100 μg/mL) in 3T3‐L1 cells (Figure 4c). Thus, these results suggest that BW extract might reduce mitochondrial activity by reducing lipolysis as well as lactic acid levels during glycolysis.

## 4 DISCUSSION

Generally, CAC is well‐known for the loss of skeletal muscle and/or fat mass (Kim, Jung, et al., 2023; Ryden & Arner, 2007; Weber et al., 2022). Patients with CAC have severe metabolic alterations, such as protein, lipid, and carbohydrate metabolism. Some studies showed that the CAC‐associated metabolic disorder would possibly be reversed by nutritional supplements (Fearon et al., 2011; Somerville & Koornneef, 2002). A few studies have demonstrated the effects of BW extract in adipocytes and high‐fat‐dieted rats on adipogenesis (Evershed, 1999; Issara et al., 2019, 2020; Sugita et al., 2013). Although BW extract treatment for 24 h decreased lipid accumulation and lipogenic‐related genes in 3T3‐L1 mature adipocytes (Issara et al., 2019), BW extract restored adipocyte differentiation and lipid aggregation, which are reduced by CCM (Figure 1). In vitro adipogenesis results showed a significant increase in lipid accumulation and adipogenic‐related gene expression during adipogenesis from preadipocytes to mature adipocytes (Figure 2). Moreover, in line with adipogenic capacity, BW extract treatment could downregulate lipolysis genes and mitochondrial OCR in fully differentiated 3T3‐L1 adipocytes (Figures 3 and 4). Based on the results presented in this study, BW extract could be used as a potential supplement in anti‐cachexia therapy and might be an alternative medicine to reverse energy imbalance.

In the aspect of active ingredients in BW, various flavonoids, such as chrysin, galangin, pinobanksin, pinocembrin, and techtochrysin, were identified in BW (Tomás‐Barberán et al., 1993). Pinobanksin and pinocembrin affect energy homeostasis by activating a free fatty acid receptor 4 involved in adipogenesis regulation (Cho et al., 2020). Galangin and pinocembrin have a combinatorial/synergistic effect on insulin sensitivity by regulating Akt/mTOR signaling (Liu et al., 2018). These compounds are likely to have a major role in the restorative effect of BW extract on cachexia‐associated adipose tissue atrophy. However, further characterization of BW extract is needed for the identification of active compounds responsible for increased adipogenesis in CAC conditions. Thus, further research is required to elucidate the detailed mechanism of action of BW extract on adipogenesis restoration under CAC conditions.

A limitation of this study is that it needs further investigation to determine whether the BW extract has the same effects with regard to adipogenesis, lipolysis, and mitochondrial oxygen consumption in the in vivo CAC model and in CAC patients as shown in 3T3‐L1 adipocytes. Also, further characterization of BW extract is needed for the identification of active compounds responsible for increased adipogenesis in CAC conditions.

## 5 CONCLUSION

In this study, BW extract showed considerable improvement in adipogenesis and lipid accumulation under CAC conditions as well as adipogenic gene expression and its target gene during normal adipogenesis. Administration of BW extract to adipocytes also decreased lipolysis. In addition, BW extract significantly downregulated the mitochondrial fatty acid oxidation‐related genes, OCR, and ECAR. Although we need further investigation into the effects of BW extract in an in vivo CAC model, our results suggest that BW extract could improve adipose atrophy in CAC through the activation of adipogenesis and inhibition of lipolysis in adipocytes.

## AUTHOR CONTRIBUTIONS

**Hyun‐Jun Jang:** Investigation (equal); writing – original draft (equal). **Hyun‐Yong Kim:** Investigation (supporting). **Ji Hyo Lyu:** Investigation (supporting). **Subramanian Muthamil:** Investigation (supporting). **Ung Cheol Shin:** Investigation (supporting). **Hyo Seon Kim:** Investigation (supporting). **Jieun Jeong:** Investigation (supporting). **Suwhan Chang:** Investigation (supporting). **Yun Kyung Lee:** Conceptualization (equal); funding acquisition (equal); investigation (equal); writing – original draft (equal); writing – review and editing (equal). **Jun Hong Park:** Conceptualization (equal); funding acquisition (equal); investigation (equal); writing – original draft (equal); writing – review and editing (equal).

## CONFLICT OF INTEREST STATEMENT

All authors declare that they have no conflict of interest.

## ETHICS STATEMENT

This study does not involve any human or animal testing.

## INFORMED CONSENT

Written informed consent was obtained from all study participants.

## Supporting information


Figure S1.

Table S1.

## Data Availability

Data available on request from the authors.

## References

[fsn34153-bib-0001] Batista, M. L., Jr. , Neves, R. X. , Peres, S. B. , Yamashita, A. S. , Shida, C. S. , Farmer, S. R. , & Seelaender, M. (2012). Heterogeneous time‐dependent response of adipose tissue during the development of cancer cachexia. The Journal of Endocrinology, 215(3), 363–373. 10.1530/JOE-12-0307 23033362

[fsn34153-bib-0002] Bing, C. , Russell, S. , Becket, E. , Pope, M. , Tisdale, M. J. , Trayhurn, P. , & Jenkins, J. R. (2006). Adipose atrophy in cancer cachexia: Morphologic and molecular analysis of adipose tissue in tumour‐bearing mice. British Journal of Cancer, 95(8), 1028–1037. 10.1038/sj.bjc.6603360 17047651 PMC2360696

[fsn34153-bib-0003] Chen, L. , Yang, Q. , Zhang, H. , Wan, L. , Xin, B. , Cao, Y. , Zhang, J. , & Guo, C. (2020). Cryptotanshinone prevents muscle wasting in CT26‐induced cancer cachexia through inhibiting STAT3 signaling pathway. Journal of Ethnopharmacology, 260, 113066. 10.1016/j.jep.2020.113066 32505837

[fsn34153-bib-0004] Cheon, S. Y. , Chung, K. S. , Roh, S. S. , Cha, Y. Y. , & An, H. J. (2017). Bee venom suppresses the differentiation of preadipocytes and high fat diet‐induced obesity by inhibiting adipogenesis. Toxins (Basel), 10(1), 9. 10.3390/toxins10010009 29295544 PMC5793096

[fsn34153-bib-0005] Cho, H. , Kim, K. , Kim, N. , Woo, M. , & Kim, H. Y. (2020). Effect of propolis phenolic compounds on free fatty acid receptor 4 activation. Food Science and Biotechnology, 29(4), 579–584. 10.1007/s10068-019-00688-4 32296569 PMC7142188

[fsn34153-bib-0006] Chung, T. H. , Yen‐Ping Kuo, M. , Chen, J. K. , & Huang, D. M. (2011). YC‐1 rescues cancer cachexia by affecting lipolysis and adipogenesis. International Journal of Cancer, 129(9), 2274–2283. 10.1002/ijc.26174 21557215

[fsn34153-bib-0007] Daas, S. I. , Rizeq, B. R. , & Nasrallah, G. K. (2018). Adipose tissue dysfunction in cancer cachexia. Journal of Cellular Physiology, 234(1), 13–22. 10.1002/jcp.26811 30078199

[fsn34153-bib-0008] Evershed, R. P. (1999). Lipids as carriers of anthropogenic signals from prehistory. Philosophical Transactions of the Royal Society, B: Biological Sciences, 354(1379), 19–31. 10.1098/rstb.1999.0357

[fsn34153-bib-0009] Farmer, S. R. (2006). Transcriptional control of adipocyte formation. Cell Metabolism, 4(4), 263–273. 10.1016/j.cmet.2006.07.001 17011499 PMC1958996

[fsn34153-bib-0010] Fearon, K. , Strasser, F. , Anker, S. D. , Bosaeus, I. , Bruera, E. , Fainsinger, R. L. , Jatoi, A. , Loprinzi, C. , Macdonald, N. , Mantovani, G. , Davis, M. , Muscaritoli, M. , Ottery, F. , Radbruch, L. , Ravasco, P. , Walsh, D. , Wilcock, A. , Kaasa, S. , & Baracos, V. E. (2011). Definition and classification of cancer cachexia: An international consensus. The Lancet Oncology, 12(5), 489–495. 10.1016/S1470-2045(10)70218-7 21296615

[fsn34153-bib-0011] Han, J. , Wang, Y. , Qiu, Y. , Sun, D. , Liu, Y. , Li, Z. , Zhou, B. , Zhang, H. , Xiao, Y. , Wu, G. , & Ding, Q. (2022). Single‐cell sequencing unveils key contributions of immune cell populations in cancer‐associated adipose wasting. Cell Discovery, 8(1), 122. 10.1038/s41421-022-00466-3 36376273 PMC9663454

[fsn34153-bib-0012] Issara, U. , Park, S. , Lee, S. , Lee, J. , & Park, S. (2020). Health functionality of dietary oleogel in rats fed high‐fat diet: A possibility for fat replacement in foods. Journal of Functional Foods, 70, 103979. 10.1016/j.jff.2020.103979

[fsn34153-bib-0013] Issara, U. , Park, S. , & Park, S. (2019). Determination of fat accumulation reduction by edible fatty acids and natural waxes in vitro. Food Science of Animal Resources, 39(3), 430–445. 10.5851/kosfa.2019.e38 31304472 PMC6612783

[fsn34153-bib-0014] Jung, M. , Kim, S. , Kim, Y. , & Kim, M. R. (2023). The efficacy of *Plantago asiatica* L. water extract on lipid metabolism in a high‐fat diet‐induced obese C57BL/6 mice. Molecular & Cellular Toxicology, 20, 399–408. 10.1007/s13273-023-00355-0

[fsn34153-bib-0015] Kim, H. Y. , Jo, M. J. , Nam, S. Y. , Kim, K. M. , Choi, M. B. , & Lee, Y. H. (2020). Evaluating the effects of honey bee (*Apis mellifera* L.) venom on the expression of insulin sensitivity and inflammation‐related genes in co‐culture of adipocytes and macrophages. Entomological Research, 50(5), 236–244. 10.1111/1748-5967.12431

[fsn34153-bib-0016] Kim, M. H. , Kim, S. J. , Kim, S. H. , Park, W. J. , & Han, J. S. (2023). *Gryllus bimaculatus*‐containing diets protect against dexamethasone‐induced muscle atrophy, but not high‐fat diet‐induced obesity. Food Science & Nutrition, 11(6), 2787–2797. 10.1002/fsn3.3257 37324877 PMC10261823

[fsn34153-bib-0017] Kim, Y. , Jung, S. , Park, G. , Shin, H. , Heo, S. C. , & Kim, Y. (2023). Beta‐carotene suppresses cancer cachexia by regulating the adipose tissue metabolism and gut microbiota dysregulation. The Journal of Nutritional Biochemistry, 114, 109248. 10.1016/j.jnutbio.2022.109248 36503110

[fsn34153-bib-0018] Lee, C. J. , Kang, M. J. , Kim, S. , Han, I. H. , & Bae, H. (2022). Screening of phytochemicals effective on relieving cancer cachexia in cisplatin‐induced in vitro sarcopenia model. Molecular & Cellular Toxicology, 18(1), 111–120. 10.1007/s13273-021-00181-2

[fsn34153-bib-0019] Lee, J. , Park, S. , Kang, M. , Chun, J. M. , Park, J. H. , & Lee, Y. K. (2023). Adipogenic effects of Ostreae Testa water extract on white adipocytes. Molecular & Cellular Toxicology, 20, 159–165. 10.1007/s13273-023-00335-4

[fsn34153-bib-0020] Lee, J. E. , Cho, Y. W. , Deng, C. X. , & Ge, K. (2020). MLL3/MLL4‐associated PAGR1 regulates adipogenesis by controlling induction of C/EBPbeta and C/EBPdelta. Molecular and Cellular Biology, 40(17), e00209‐20. 10.1128/MCB.00209-20 32601106 PMC7431048

[fsn34153-bib-0021] Lee, S. G. , Chae, J. , Woo, S. M. , Seo, S. U. , Kim, H. J. , Kim, S. Y. , Schlaepfer, D. D. , Kim, I. S. , Park, H. S. , Kwon, T. K. , & Nam, J. O. (2023). TGFBI remodels adipose metabolism by regulating the Notch‐1 signaling pathway. Experimental & Molecular Medicine, 55(3), 520–531. 10.1038/s12276-023-00947-9 36854775 PMC10073093

[fsn34153-bib-0022] Lee, S. G. , Kim, N. , Kim, S. M. , Park, I. B. , Kim, H. , Kim, S. , Kim, B. G. , Hwang, J. M. , Baek, I. J. , Gartner, A. , Park, J. H. , & Myung, K. (2020). Ewing sarcoma protein promotes dissociation of poly(ADP‐ribose) polymerase 1 from chromatin. EMBO Reports, 21(11), e48676. 10.15252/embr.201948676 33006225 PMC7645264

[fsn34153-bib-0023] Lee, S.‐G. , Kim, N. , Park, I. B. , Park, J. H. , & Myung, K. (2021). Tissue‐specific DNA damage response in mouse whole‐body irradiation. Molecular & Cellular Toxicology, 18(1), 131–139. 10.1007/s13273-021-00195-w

[fsn34153-bib-0024] Lee, Y. H. , Jang, H. J. , Kim, S. , Choi, S. S. , Khim, K. W. , Eom, H. J. , Hyun, J. , Shin, K. J. , Chae, Y. C. , Kim, H. , Park, J. , Park, N. H. , Woo, C. Y. , Hong, C. H. , Koh, E. H. , Nam, D. , & Choi, J. H. (2021). Hepatic MIR20B promotes nonalcoholic fatty liver disease by suppressing PPARA. eLife, 10, e70472. 10.7554/eLife.70472 34964438 PMC8758141

[fsn34153-bib-0025] Lee, Y. K. , Sohn, J. H. , Han, J. S. , Park, Y. J. , Jeon, Y. G. , Ji, Y. , Dalen, K. T. , Sztalryd, C. , Kimmel, A. R. , Kim, J. B. , & Kim, J. B. (2018). Perilipin 3 deficiency stimulates thermogenic beige adipocytes through PPARalpha activation. Diabetes, 67(5), 791–804. 10.2337/db17-0983 29440067 PMC5909993

[fsn34153-bib-0026] Liu, Y. , Liang, X. , Zhang, G. , Kong, L. , Peng, W. , & Zhang, H. (2018). Galangin and pinocembrin from propolis ameliorate insulin resistance in HepG2 cells via regulating Akt/mTOR signaling. Evidence‐based Complementary and Alternative Medicine, 2018, 7971842. 10.1155/2018/7971842 30420897 PMC6215570

[fsn34153-bib-0027] Lopes, M. A. , Oliveira Franco, F. , Henriques, F. , Peres, S. B. , & Batista, M. L., Jr. (2018). LLC tumor cells‐derivated factors reduces adipogenesis in co‐culture system. Heliyon, 4(7), e00708. 10.1016/j.heliyon.2018.e00708 30094378 PMC6071679

[fsn34153-bib-0028] Nakano, T. , Sasaki, Y. , Norikura, T. , Hosokawa, Y. , Kasano, M. , Matsui‐Yuasa, I. , Huang, X. , Kobayashi, Y. , & Kojima‐Yuasa, A. (2023). The suppression of the differentiation of adipocytes with *Mallotus furetianus* is regulated through the posttranslational modifications of C/EBPbeta. Food Science & Nutrition, 11(10), 6151–6163. 10.1002/fsn3.3551 37831750 PMC10563708

[fsn34153-bib-0029] Palesty, J. A. , & Dudrick, S. J. (2003). What we have learned about cachexia in gastrointestinal cancer. Digestive Diseases, 21(3), 198–213. 10.1159/000073337 14571093

[fsn34153-bib-0030] Pantoja, C. , Huff, J. T. , & Yamamoto, K. R. (2008). Glucocorticoid signaling defines a novel commitment state during adipogenesis in vitro. Molecular Biology of the Cell, 19(10), 4032–4041. 10.1091/mbc.E08-04-0420 18653467 PMC2555927

[fsn34153-bib-0031] Rosen, E. D. , Walkey, C. J. , Puigserver, P. , & Spiegelman, B. M. (2000). Transcriptional regulation of adipogenesis. Genes & Development, 14, 1293. 10.1101/gad.14.11.1293 10837022

[fsn34153-bib-0032] Ryden, M. , & Arner, P. (2007). Fat loss in cachexia‐‐is there a role for adipocyte lipolysis? Clinical Nutrition, 26(1), 1–6. 10.1016/j.clnu.2006.09.009 17095126

[fsn34153-bib-0033] Somerville, C. , & Koornneef, M. (2002). A fortunate choice: The history of *Arabidopsis* as a model plant. Nature Reviews. Genetics, 3(11), 883–889. 10.1038/nrg927 12415318

[fsn34153-bib-0034] Sugita, J. , Yoneshiro, T. , Hatano, T. , Aita, S. , Ikemoto, T. , Uchiwa, H. , Iwanaga, T. , Kameya, T. , Kawai, Y. , & Saito, M. (2013). Grains of paradise (*Aframomum melegueta*) extract activates brown adipose tissue and increases whole‐body energy expenditure in men. The British Journal of Nutrition, 110(4), 733–738. 10.1017/S0007114512005715 23308394

[fsn34153-bib-0035] Sun, X. , Feng, X. , Wu, X. , Lu, Y. , Chen, K. , & Ye, Y. (2020). Fat wasting is damaging: Role of adipose tissue in cancer‐associated cachexia. Frontiers in Cell and Development Biology, 8, 33. 10.3389/fcell.2020.00033 PMC7028686 32117967

[fsn34153-bib-0036] Tang, Q. Q. , Zhang, J. W. , & Daniel Lane, M. (2004). Sequential gene promoter interactions by C/EBPbeta, C/EBPalpha, and PPARgamma during adipogenesis. Biochemical and Biophysical Research Communications, 318(1), 213–218. 10.1016/j.bbrc.2004.04.017 15110775

[fsn34153-bib-0037] Tomás‐Barberán, F. , Ferreres, F. , Tomás‐Lorente, F. , & Ortiz, A. (1993). Flavonoids from *Apis mellifera* beeswax. Zeitschrift für Naturforschung. Section C, 48(1–2), 68–72. 10.1515/znc-1993-1-213

[fsn34153-bib-0038] Wang, Q. A. , Tao, C. , Gupta, R. K. , & Scherer, P. E. (2013). Tracking adipogenesis during white adipose tissue development, expansion and regeneration. Nature Medicine, 19(10), 1338–1344. 10.1038/nm.3324 PMC4075943 23995282

[fsn34153-bib-0039] Weber, B. Z. C. , Arabaci, D. H. , & Kir, S. (2022). Metabolic reprogramming in adipose tissue during cancer cachexia. Frontiers in Oncology, 12, 848394. 10.3389/fonc.2022.848394 35646636 PMC9135324

[fsn34153-bib-0040] Yan, B. , Li, X. , Peng, M. , Zuo, Y. , Wang, Y. , Liu, P. , Ren, W. , & Jin, X. (2023). The YTHDC1/GLUT3/RNF183 axis forms a positive feedback loop that modulates glucose metabolism and bladder cancer progression. Experimental & Molecular Medicine, 55(6), 1145–1158. 10.1038/s12276-023-00997-z 37258572 PMC10318083

[fsn34153-bib-0041] Yang, A. , & Mottillo, E. P. (2020). Adipocyte lipolysis: From molecular mechanisms of regulation to disease and therapeutics. The Biochemical Journal, 477(5), 985–1008. 10.1042/BCJ20190468 32168372 PMC7187988

