# Supplementary figures and images for: Bee (Apis mellifera L. 1758) wax restores adipogenesis and lipid accumulation of 3T3‐L1 cells in cancer‐associated cachexia condition
Source: Food Sci Nutr. 2024 Apr 17;12(7):5027–35. doi: 10.1002/fsn3.4153 (PMC11266878; doi:10.1002/fsn3.4153)

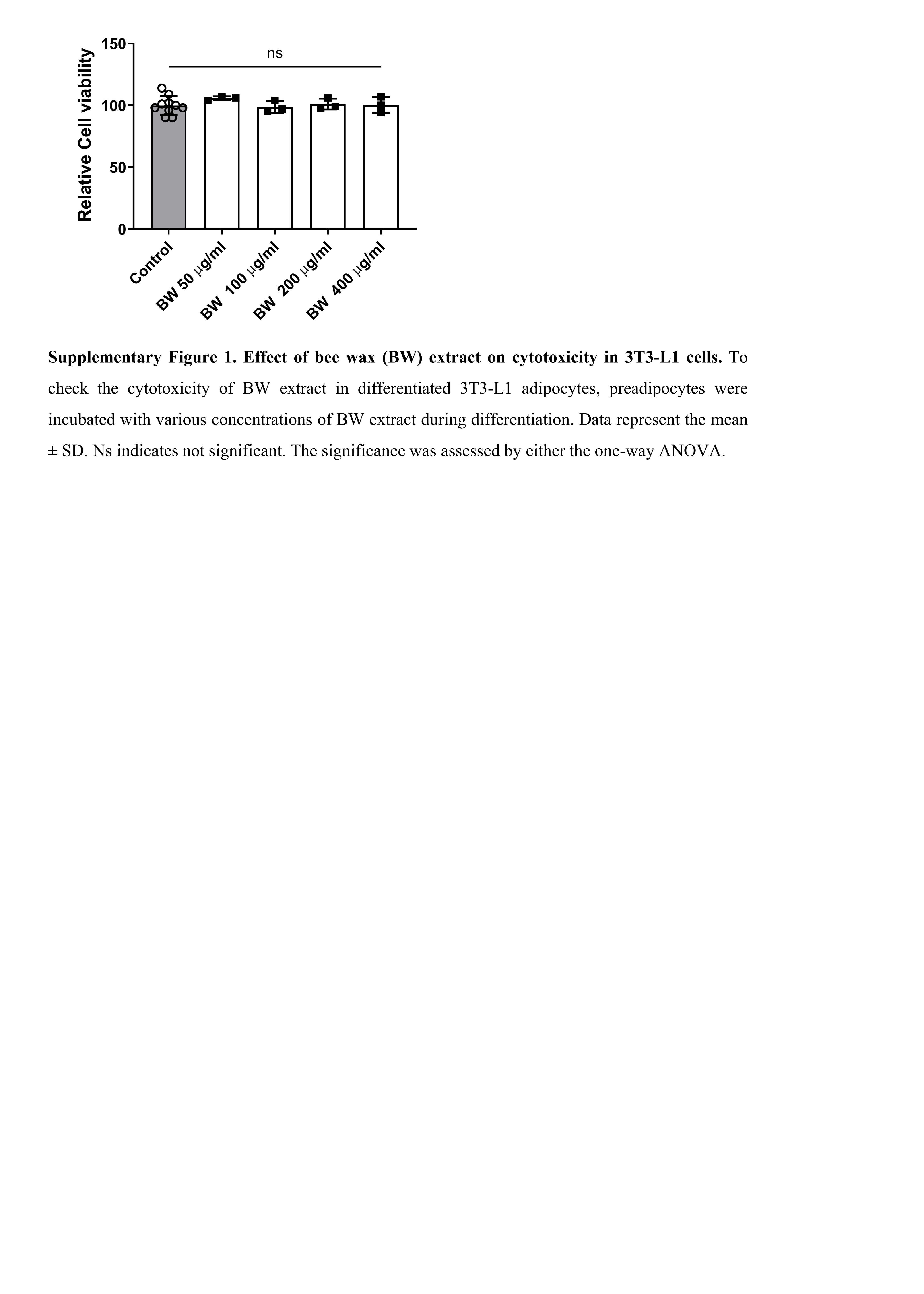

Supplement: Supplementary file 1 — Figure S1. [file FSN3-12-5027-s002.jpeg]
