# Supplementary material for: Bee (Apis mellifera L. 1758) wax restores adipogenesis and lipid accumulation of 3T3‐L1 cells in cancer‐associated cachexia condition
Source: Food Sci Nutr. 2024 Apr 17;12(7):5027–35. doi: 10.1002/fsn3.4153 (PMC11266878; doi:10.1002/fsn3.4153)
Supplement: Supplementary file 2 — Table S1. [file FSN3-12-5027-s001.docx]

**Supplementary Table S1. Primer used in quantitative real-time RT-PCR (qRT-PCR)**

|  | Forward (5′ to 3′) | Reverse (5′ to 3′) |
| --- | --- | --- |
| PPARγ | TTGCTGAACGTGAAGCCCATCGAGG | GTCTTGTAGATCTCCTGGAGCAG |
| C/EBPα | GCGGGAACGCAACAACATC | GTCACTGGTCAACTCCAGCAC |
| aP2 | AAGGTGAAGAGCATCATAACCCT | TCACGCCTTTCATAACACATTCC |
| ATGL | TCCGAGAGATGTGCAAACAG | CTCCAGCGGCAGAGTATAGG |
| HSL | GCGCTGGAGGAGTGTTTTT | CCGCTCTCCAGTTGAACC |
| CPT1α | ACTCCGCTCGCTCATTCCG | CACACCCACCACCACGATAA |
| Acox1 | CAGGAAGAGCAAGGAAGTGG | CCTTTCTGGCTGATCCCATA |
| Cox8b | TGCGAAGTTCACAGTGGTTC | ATGCTGCGGAGCTCTTTTTA |
| Cyclophilin | CAGACGCCACTGTCGCTTT | TGTCTTTGGAACTTTGTGTG |
